# Nutrient metabolism in the liver and muscle of juvenile blunt snout bream (*Megalobrama amblycephala*) in response to dietary methionine levels

**DOI:** 10.1038/s41598-021-03084-3

**Published:** 2021-12-13

**Authors:** Ke Ji, Hualiang Liang, Mingchun Ren, Xianping Ge, Liangkun Pan, Heng Yu

**Affiliations:** 1 Wuxi Fisheries College, Nanjing Agricultural University, Wuxi, 214081 China; 2 Key Laboratory for Genetic Breeding of Aquatic Animals and Aquaculture Biology, Freshwater Fisheries Research Center (FFRC), Chinese Academy of Fishery Sciences (CAFS), Wuxi, 214081 China

**Keywords:** Biochemistry, Molecular biology

## Abstract

A 75-day rearing trial was designed to study the response of juvenile *Megalobrama amblycephala* to dietary methionine (Met) levels. Three practical diets with graded Met levels (0.40%, 0.84% and 1.28% dry matter) were prepared to feed the juvenile fish. The results showed that the 0.84% Met diet significantly improved the growth compared with 0.40% diets. Compared with 0.84% and 1.28% Met, 0.40% Met significantly increased the hepatic lipid content, while decreasing the muscular lipid and glycogen contents. 0.40% Met decreased the protein levels of phospho-Eukaryotic initiation factor 4E binding protein-1 (p-4e-bp1), 4e-bp1 and Ribosomal protein S6 kinase 1 in the liver, compared with 0.84% diet, while an increasing trend was observed in the muscle. Met supplementation tended to decrease and increase lipid synthesis in the liver and muscle, respectively, via changing mRNA levels of *sterol regulatory element-binding protein 1*, *fatty acid synthetase* and *acetyl-CoA carboxylase*. 1.28% dietary Met promoted fatty acid β-oxidation and lipolysis in both the liver and muscle by increasing *carnitine palmitoyl transferase 1*, *peroxisome proliferator activated receptor alpha*, *lipoprotein lipase* and *lipase* mRNA levels. Compared with 0.40% and 0.84% dietary Met, 1.28% Met enhanced the mRNA levels of hepatic gluconeogenesis related genes *phosphoenolpyruvate carboxykinase* (*pepck*), and *glucose-6-phosphatase*, and muscular glycolysis related genes *phosphofructokinase* (*pfk*), and *pyruvate kinase* (*pk*). The mRNA levels of hepatic *pfk*, *pk* and *glucokinase* were markedly downregulated by 1.28% Met compared with 0.84% level. Muscular *pepck*, *glycogen synthase*, and hepatic *glucose transporters 2* mRNA levels were induced by 1.28% Met. Generally, deficient Met level decreased the growth of juvenile *Megalobrama amblycephala*, and the different nutrient metabolism responses to dietary Met were revealed in the liver and muscle.

## Introduction

Blunt snout bream, *Megalobrama amblycephala*, is the main freshwater aquaculture species in China. Our previous studies have confirmed that dietary methionine (Met) is important for the growth performance of blunt snout bream, and optimal dietary Met could improve the immunity and antioxidant capacity of dietary Met of blunt snout bream^1–3^. In addition, Met has been shown to regulate glucose and lipid metabolism in fish. The primary muscle cells of turbot (*Scophthalmus maximus* L.) under Met deprivation showed inhibited expression of the key target of rapamycin (TOR) pathway elements, and genes related to glycolysis and fatty acid synthesis, while inducing fatty acid β-oxidation^4^. In cobia (*Rachycentron canadum*), Met deficiency suppressed hepatic lipogenesis related gene (*sterol regulatory element-binding protein* (*srebp1*) and *fatty acid synthetase* (*fas*)) mRNA expressions and upregulated fatty acid oxidation-related gene (*carnitine palmitoyl transferase 1* (*cpt1*), *peroxisome proliferator activated receptor alpha* (*pparα*), and *lipoprotein lipase* (*lpl*)), *phosphoenolpyruvate carboxykinase* (*pepck*) relative mRNA expression levels^5,6^. Moreover, the nutrient metabolism response to dietary Met showed species-specific responses. Rainbow trout (*Oncorhynchus mykiss*) fed Met-deficient diets showed a positive relationship with hepatic *fas* mRNA expression, and negative with *cpt1* and *fructose-1,6-biphosphatase* (*fbp*) relative mRNA expression levels^7^. While Met deficiency and excess diet induced hepatic lipid accumulation in yellow catfish (*pelteobagrus fulvidraco*)^8^. The increased lipid content in the liver and whole-body were observed in *Takifugu rubripes* fed with increasing dietary Met levels, which contradicted with hepatic relative mRNA expressions: decreased related lipogenic genes expression (*fas*, *glycerol-3-phosphate acyltransferase*, *pparγ*, *atp citrate lyase*, and *delta-9-desaturase 1*) and increased lipolytic genes expression (*acyl-CoA oxidase 1* (*acox1*), *hormone-sensitive lipase*, and *apolipoprotein b100*)^9^. These data revealed that the response of aquaculture fish to dietary Met is complex. However, the effects of dietary Met levels on nutrient metabolism of blunt snout bream are still uncertain due to the limitation of the study.

The liver and muscle are important nutrient metabolism organs in animals. In fish, the liver and muscle are both main sites of protein synthesis, lipid synthesis and excess carbohydrate storage^10,11^. Furthermore, it was found that the metabolic polytrophic response of fish species to some dietary nutrients is tissue-specific. Kolditz et al.^12^ reported that the high-energy diet repressed the activity of the lipogenic enzymes and stimulated enzymes involved in fatty acid oxidation and glycolysis in the liver of rainbow trout but had little effect on related enzymatic activities in muscle. Met-restricted feeding significantly increased the liver fat content but decreased the muscle fat content of rainbow trout^13^. The juvenile Nile tilapia (*Oreochromis niloticus*) fed on diets containing 10 mg/kg clenbuterol exhibited decreased hepatic *fas* relative mRNA expression level but had increased *fas* relative mRNA expression level in the muscle^14^. However, in blunt snout bream, a lot of studies reported that hepatic nutrient metabolism was a response to a dietary composition including amino acids (except Met)^15–18^. Unlike the liver, there are few studies that have investigated the effect in muscle, and many of these researches focused on muscle development, composition rather than nutrient metabolism^19,20^. The response of the nutrient metabolism in the liver and the muscle to dietary administration is still unclear.

Therefore, the current study was designed to investigate the effects of dietary Met levels on the nutrient metabolism of *Megalobrama amblycephala* and to study the different metabolic responses in the liver and muscle.

## Results

### The growth performance

As shown in Table 1, final body weight (FBW), weight gain rate (WGR) and specific growth rate (SGR) of the fish fed Met-supplemented diets (0.84% and 1.28% dietary Met) were significantly increased compared with those in fish fed 0.40% diet (*P* < 0.05). FBW, SGR and WGR were significantly lowered in the 0.40% Met diet group than those in fish fed the 0.84% Met diet (*P* < 0.05). The values of feed conversion ratio (FCR) showed the opposite trend.

**Table 1:** The effects of dietary methionine levels on the growth performance of juvenile blunt snout bream (*Megalobrama amblycephala*) (means ± SEM)^1^.

Dietary Met levels (%)	IBW^2^ (g)	FBW^3^ (g)	FCR^4^	SGR^5^ (%/day)	WGR^6^ (%)
0.40	4.38 ± 0.01	44.60 ± 0.17^a^	0.92 ± 0.02^b^	3.10 ± 0.01^a^	918.97 ± 6.33^a^
0.84	4.35 ± 0.01	51.41 ± 0.25^c^	0.82 ± 0.01^a^	3.29 ± 0.01^c^	1081.02 ± 5.85^c^
1.28	4.37 ± 0.01	46.90 ± 0.40^b^	0.87 ± 0.01^ab^	3.17 ± 0.01^b^	977.55 ± 9.81^b^

^1^All data are mean value of three replicates ± SEM (n = 3). Means in the same column with different superscripts “a, b, c” are significantly different (*P* < 0.05).

^2^*IBW*: initial body weight.

^3^*FBW*: final body weight.

^4^Feed conversion ratio (FCR).

^5^Specific growth rate (SGR, %/day).

^6^Weight gain rate (WGR, %).

### Composition of whole body and tissues

As shown in Table 2, there were no marked differences in the whole body composition (moisture, crude protein, lipid, and ash) contents among the fish fed the three practical diets (*P* > 0.05). Lipid content in fish fed 0.40% Met diet was significantly higher than that in the fish fed other diets (*P* < 0.05). While 0.40% Met level significantly decreased the lipid content in the muscle compared with 0.84% and 1.28% Met levels (*P* < 0.05).

**Table 2:** Effects of dietary methionine levels on whole body composition of blunt snout bream (*Megalobrama amblycephala*) (Means ± SEM)^1^.

Index	Dietary Met levels (%)		
	0.40	0.84	1.28
**Proximate composition of whole body (% wet weight)**			
Moisture	73.05 ± 0.25	73.64 ± 0.22	73.61 ± 0.05
Protein	16.30 ± 0.10	16.67 ± 0.20	16.53 ± 0.11
Lipid	6.56 ± 0.05	6.27 ± 0.04	6.27 ± 0.11
Ash	3.49 ± 0.16	3.21 ± 0.04	3.43 ± 0.03
**Lipid contents in tissues (% wet weight)**			
Liver	5.02 ± 0.41^b^	3.65 ± 0.31^a^	3.72 ± 0.27^a^
Muscle	0.52 ± 0.05^a^	0.81 ± 0.06^b^	0.87 ± 0.06^b^

^1^All data are mean value of three replicates ± SEM (n = 3). Means in the same column with different superscripts “a, b” are significantly different (*P* < 0.05).

### The plasma parameters

**Figure 1:**
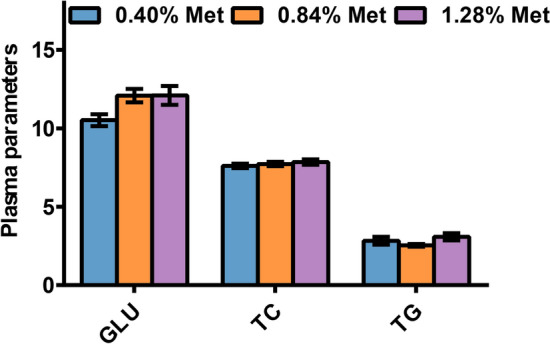
The plasma parameters in juvenile *Megalobrama amblycephala* fed grade methionine level diets. Data are expressed as means with SEM (n = 9). Values with different letters in lower case are significantly different (*P* < 0.05). *GLU* glucose, *TC* total triglyceride, *TG* total cholesterol.

As indicated in Fig. 1, the dietary Met levels did not significantly affect the levels of plasma glucose (GLU), total cholesterol (TC), and total triglyceride (TG) (*P* > 0.05), although GLU contents tended to increase with increasing dietary Met levels.

### The glycogen contents in the liver and muscle

**Figure 2:**
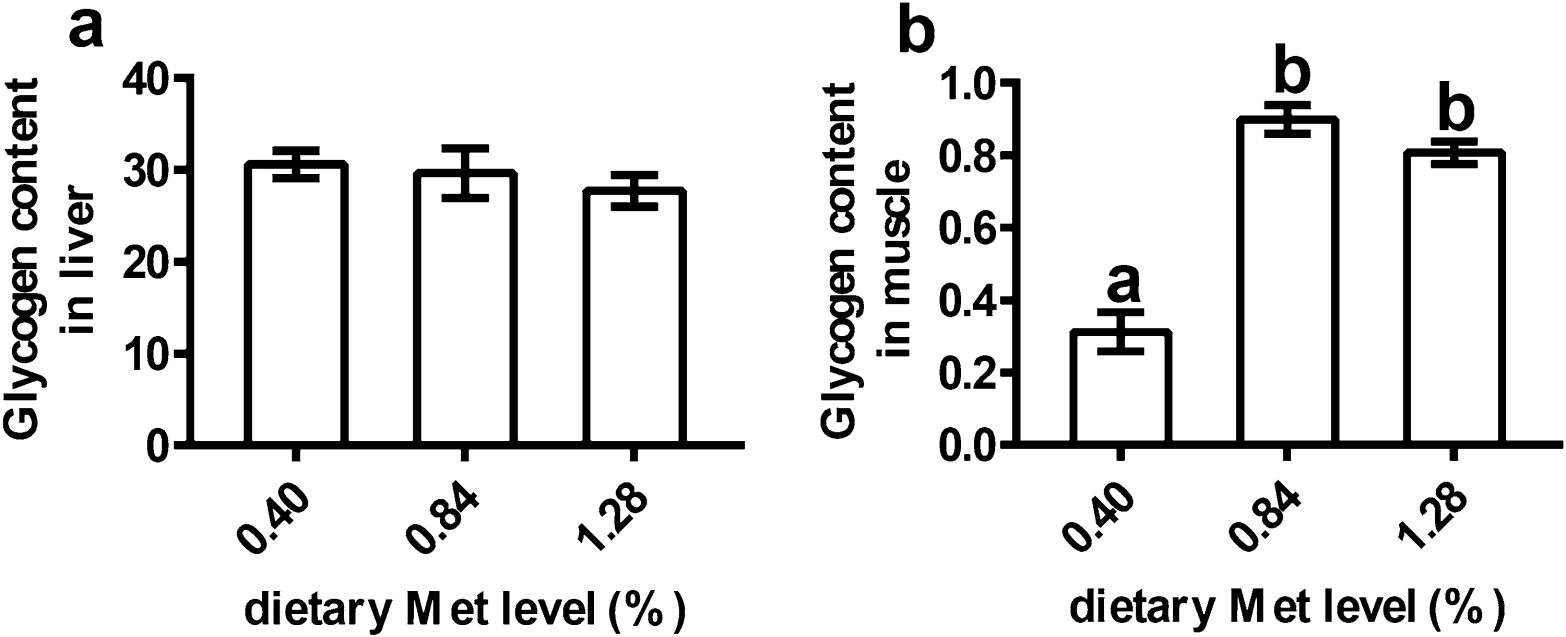
The glycogen contents in the liver and muscle of juvenile *Megalobrama amblycephala* fed grade methionine level diets. Data are expressed as means with SEM (n = 12). Values with different letters in lower case are significantly different (*P* < 0.05).

As shown in Fig. 2, the dietary Met levels had no significant effects on the glycogen contents in the liver (*P* > 0.05). However, 0.84% and 1.28% Met levels significantly increased muscular glycogen contents compared with 0.40% Met diet (*P* < 0.05).

### The protein and gene expression levels of the TOR signaling pathway in the liver and muscle

**Figure 3:**
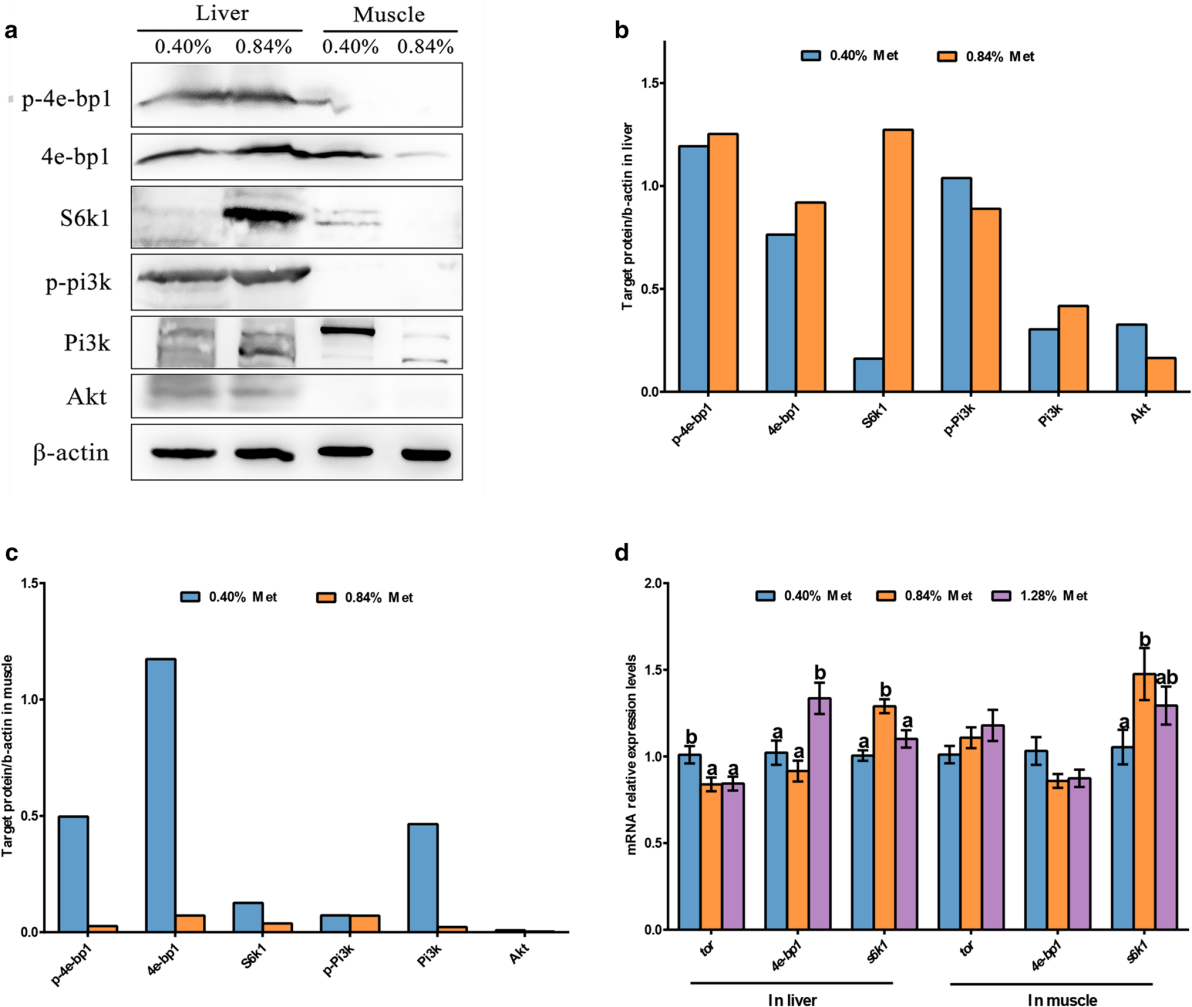
TOR and PI3K signaling in the liver and muscle of juvenile *Megalobrama amblycephala* fed grade methionine level diets. The protein levels and/or phosphorylation of 4e-bp1, S6k1, Pi3k and Akt were examined by western blots (**a**) and quantitated in the liver (**b**) and muscle (**c**). mRNA levels of TOR signaling were quantified in the liver and muscle (**d**). Data are expressed as means with SEM (n = 12). Values with different letters in lower case are significantly different (*P* < 0.05). The blots were cut prior to hybridization with antibodies, original blots are presented in Supplementary Figs. S1–S7. *tor*, *target of rapamycin*; 4e-bp1, eukaryotic initiation factor 4e binding protein-1; S6k1, ribosomal protein s6 kinase 1; Pi3k, phosphatidylinositol 3-kinase; Akt, protein kinase b.

As shown in Fig. 3a,b, the protein levels of p-4e-bp1, 4e-bp1, S6k1 and Pi3k in the liver of fish fed the 0.84% Met diet were higher than those in fish fed the 0.40% diet. The hepatic protein levels of p-PI3K and Akt in the 0.40% Met group were higher than those in the 0.84% group (Fig. 3b). Contrary to the liver, the protein levels of p-4e-bp1, 4e-bp1, S6k1, Pi3k in the muscle of fish fed the 0.40% Met diet were higher than those in fish fed 0.84% diet; and the p-Pi3k and Akt protein levels in 0.40% and 0.84% diets were similar, and lower than those in the liver (Fig. 3c).

The mRNA expression levels were shown in Fig. 3d. Compared with 0.84% dietary Met, 0.40% Met significantly promoted hepatic *tor* mRNA levels, while hepatic *4e-bp1* mRNA levels were significantly upregulated by 1.28% Met level (*P* < 0.05). The relative mRNA levels of *s6k1* in both the liver and muscle were markedly downregulated by 0.40% dietary Met compared with 0.84% Met level (*P* < 0.05). Dietary Met levels had no significant effects on muscular *tor* or *4e-bp1* mRNA expressions (*P* > 0.05).

### The expression of lipid metabolism related genes in the liver and muscle

**Figure 4:**
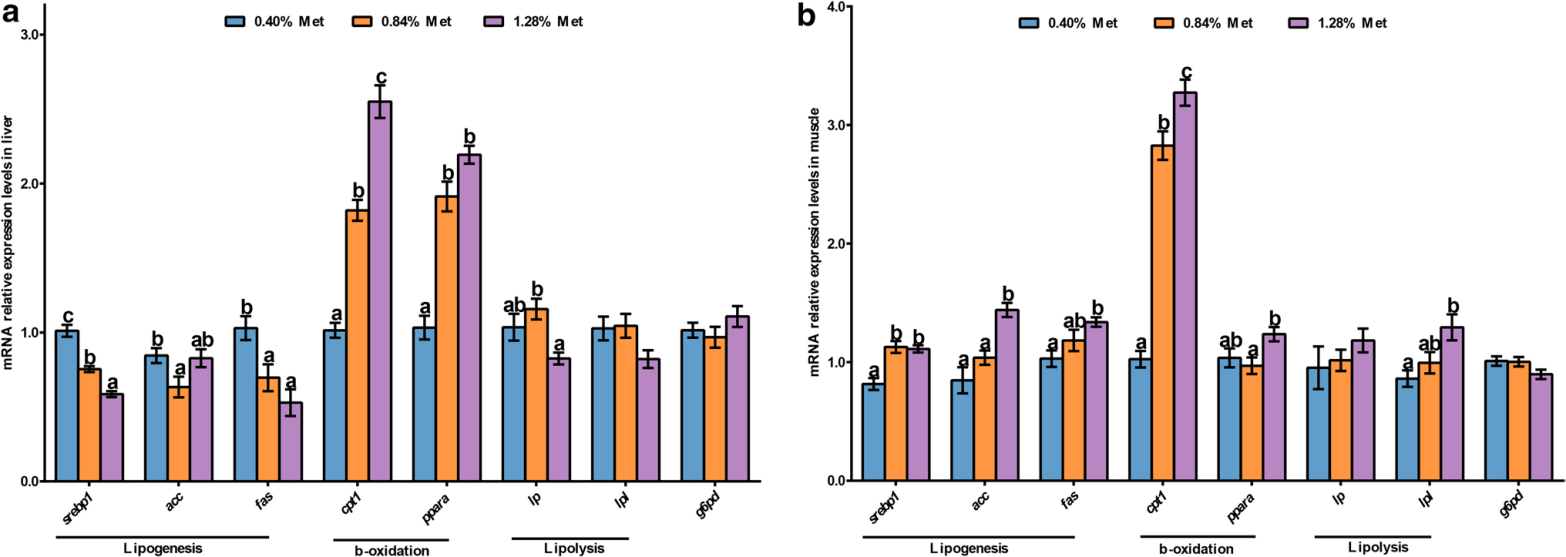
Lipid metabolism gene mRNA levels in the liver (**a**) and muscle (**b**) of juvenile *Megalobrama amblycephala* fed graded methionine level diets. Data are expressed as means with SEM (n = 12). Values with different letters in lower case are significantly different (*P* < 0.05). *srebp1*, *sterol regulatory element-binding protein 1*; *acc*, *acetyl-coa carboxylase*; *fas*, *fatty acid synthetase*; *cpt1*, *carnitine palmitoyl transferase 1*; *pparα*, *peroxisome proliferator activated receptor alpha*; *lp*, *lipase*; *lpl*, *lipoprotein lipase*; *g6pd*, *glucose-6-phosphate dehydrogenase*.

As presented in Fig. 4a, hepatic *srebp1*, *fas* and *acetyl-CoA carboxylase* (*acc*) mRNA levels were induced by 0.40% dietary Met level (*P* < 0.05). Hepatic *cpt1* and *pparα* mRNA levels were increased with increasing dietary Met levels (*P* < 0.05). Dietary Met levels of 1.28% significantly reduced hepatic *lipase* (*lp*) mRNA expression levels compared with 0.84% Met level (*P* < 0.05). While in the muscle, the mRNA levels of *srebp1*, *fas*, *acc*, *cpt1*, *pparα* and *lpl* were significantly upregulated in fish fed 1.28% Met diet, compared with 0.40% or 0.84% Met diet (*P* < 0.05) (Fig. 4b). There were no significant differences in the mRNA expression levels of muscular *lp* and hepatic *lpl* in fish fed the graded Met level diets (*P* > 0.05) (Fig. 4a,b). And the mRNA levels of *glucose-6-phosphate dehydrogenase* (*g6pd*) in both the liver and muscle were not significantly affected by dietary Met levels (*P* > 0.05).

### The expression of glucose metabolism related genes in the liver and muscle

**Figure 5:**
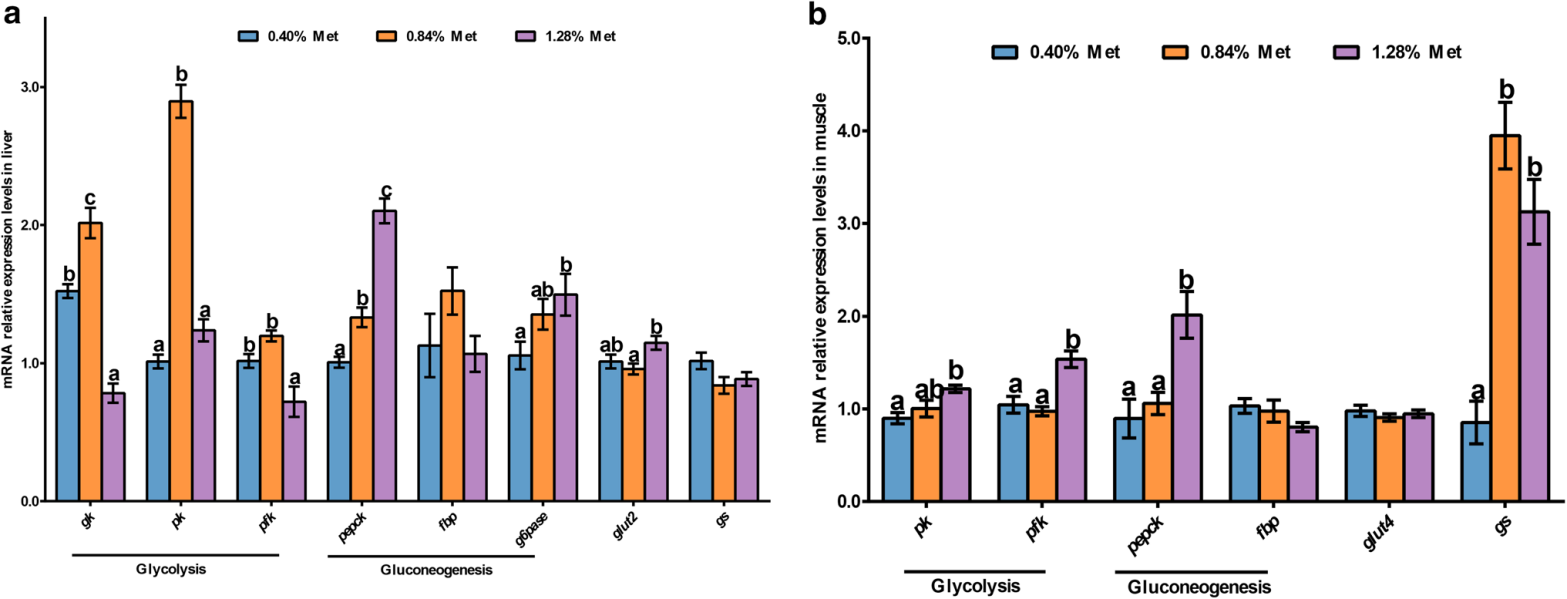
Glucose metabolism related genes mRNA levels in the liver (**a**) and muscle (**b**) of juvenile *Megalobrama amblycephala* fed graded methionine diets. Data are expressed as means with SEM (n = 12). Values with different letters in lower case are significantly different (*P* < 0.05). *gk*, *glucokinase*; *pk*, *pyruvate kinase*; *pfk*, *phosphofructokinase*; *pepck*, *phosphoenolpyruvate carboxykinase*; *fbp*, *fructose-1,6-biphosphatase*; *g6pase*, *glucose-6-phosphatase*; *glut2*, *glucose transporters 2*; *glut4*, *glucose transporters 4*; *gs*, *glycogen synthase.*

As presented in Fig. 5a,b, the mRNA expression levels of hepatic *pepck* and *glucose-6-phosphatase* (*g6pase*), and muscular *pepck*, *phosphofructokinase* (*pfk*), *pyruvate kinase* (*pk*) and *glycogen synthase* (*gs*) were markedly increased by 1.28% Met diet compared with the 0.40% diet (*P* < 0.05). *gk*, *pfk* and *pk* mRNA levels in the liver were significantly suppressed by 1.28% diet compared with 0.84% diets (*P* < 0.05). *Glucose transporters 2* (*glut2*) mRNA levels in the liver were significantly induced by 1.28% Met diet compared to 0.84% diet (*P* < 0.05); while muscular *glut4* and *fbp*, and hepatic *gs* and *fbp* mRNA levels were not markedly affected by dietary Met levels (*P* > 0.05).

## Discussion

### Optimal dietary Met levels improved the growth performance of juvenile *Megalobrama amblycephala*

The optimal Met levels in diets could improve the growth performance of fish, which has been proven in many fish species, such as large yellow croaker, *Pseudosciaena crocea* R^21^; juvenile humpback grouper, *Cromileptes altivelis*^22^; blunt snout bream^1^; Chinese sucker*, Myxocyprinus asiaticus*^23^; and grass carp, *Ctenopharyngodon idella*^24^. Similarly, in the present study, the data related to growth performance indicated that Met supplementation (0.84% and 1.28% dietary Met) significantly improved FBW, WGR and SGR of juvenile blunt snout bream compared with the diet without Met supplementation (0.40% Met diet). This result was consistent with the conclusion in Liao et al.^1^ that dietary Met played an important role in the growth performance of juvenile *Megalobrama amblycephala*. In our present study, the juvenile fish fed diets containing graded dietary Met levels showed no significant difference in whole body composition. Similar results were also reported in large yellow croaker and juvenile cobia^21,25^. In addition, although the slight change in body protein and lipid contents had a similar trend with the results observed in Liao et al.^1^, which found the significant effects on body protein and lipid contents in response to dietary Met level. The reason maybe partly attributed to the different culture environment. A similar comparison result was also observed in cobia fed dietary Met diets^6,25^. In other related studies, some studies revealed that optimal dietary Met levels markedly increased crude protein and decreased crude lipid content in whole body composition such as in Chinese sucker and Indian major carp (*Cirrhinus mrigala*)^1,26^. Whereas Yan et al.^27^ reported that whole body protein and lipid contents were significantly increased with increasing dietary Met up to 1.58% in juvenile rockfish (*Sebastes schlegeli*). These results suggested that the various change of whole body composition of fish species in response to dietary Met maybe species-specific, due to the different metabolic systems of fish species. However, the mechanism of the specific-metabolism response to dietary Met is unclear, and needs to be further investigated. In addition, 0.40% Met level significantly increased the lipid content in the liver compared with high Met diets. A Met-deficient diet was also found to increase the hepatic lipid content of broiler^28^ and *P. fulvidraco*^8^. In the present study, it was observed that the lipid content in the muscle was very low (< 1%), and 0.40% dietary Met level as well decreased lipid content in muscle. Therefore, the lack of dramatic difference in crude lipid content of body composition might be related to the differences in lipid accumulation in tissues, which also implied that the tissue-specific metabolism in blunt snout bream fed graded methionine diets.

### 0.40% dietary Met decreased hepatic TOR signaling, while improved muscular TOR signaling in juvenile *Megalobrama amblycephala*

The present study also investigated the response of TOR pathway related protein synthesis to dietary Met. 0.40% dietary Met level  decreased TOR signaling in the liver of blunt snout bream; this was evidenced by reduced protein levels of hepatic S6k1 and p-4e-bp1, the downstream of TOR that regulate protein synthesis^29^, in the 0.40% diet group. This result indicated that the liver of blunt snout bream is sensitive to Met via TOR pathway, and 0.84% Met diet could promote hepatic protein synthesis compared with the 0.40% diet. Before this study, Dai et al.^30^ reported that trout hepatocytes treated with four-fold amino acids (4 × AA) combined with insulin significantly activated the TOR pathway compared with the control. The present study was also consistent with the finding that TOR pathway key genes in porcine mammary epithelial cells were significantly increased by a mix of d- and l-Met compared with no Met^31^. However, TOR pathway response to dietary Met in blunt snout bream showed a tissue-specific and dose-dependent response in this study. Increased protein levels of S6k1, 4e-bp1 and p-4e-bp1 in the muscle of blunt snout bream were observed in the 0.40% Met diet but not in the 0.84% diet that is similar to the liver. Additionally, the trends in the mRNA expression levels of TOR pathway key genes were different from the trends at the protein level. Similar phenomena were also observed in the study by Zeitz et al.^32^, which may be due to the temporal and spatial differences between transcription and translation.

### 0.40% dietary Met increased hepatic lipid accumulation related genes expression, while suppressed lipogenesis in the muscle of juvenile *Megalobrama amblycephala*

Hepatic *srebp1*, *acc* and *fas* mRNA expression levels were markedly induced by 0.40% dietary Met compared with 0.84% or 1.28% Met levels, which was consistent with the result in lipid content of liver. SREBP1 is an important nuclear transcription factor in lipid synthesis, that controls the synthesis of enzymes involved in ACC and FAS^33,34^. Met restriction enhanced whole lipogenic capacities of growing pigs^35,36^. The current results might indicate that low Met (0.40%) promoted liver lipid synthesis via increasing related genes in blunt snout bream. Similar results were also found in other fish species. In Atlantic salmon (*Salmo salar*), Met deficiency contributed to high Fas activity and triglyceride accumulation in the liver^37^. Met deficiency also induced *fas* and *srebp1* expression in rainbow trout^38^. Recent studies report that PI3K/Akt activates SREBPs, major transcriptional regulators of lipid metabolism^39,40^. Akt activation was reported to be the necessary and sufficient factor for the increase of SREBP1C and lipid accumulation in the liver^41,42^. Yecies et al.^43^ found that Akt could induce hepatic SREBP1C and lipogenesis via parallel mTORC1-dependent and mTORC1-independent pathways. In the current study, the protein levels of p-Pi3k and Akt in the liver were increased by 0.40% Met, consistent with *srebp1* but not *tor*, which might imply that low dietary Met level (0.40%) potentially increased hepatic lipid accumulation in a Pi3k/Akt-*srebp1* independent TOR manner.

In contrast to the lipid synthesis promoted by 0.40% Met in the liver, high dietary Met levels (0.84% and 1.28%) tended to promote lipogenesis in the muscle in this study. The evidence was that the mRNA levels of muscular lipogenesis genes including *srebp1*, *acc*, and *fas*, as well as the lipid content in the muscle, were markedly induced by 0.84% and 1.28% dietary Met. The results were in line with turbot primary muscle cells treated with Met deprivation, which significantly reduced the relative mRNA expression of *fas* and *srebp1* compared to those in the control group^4^. Latimer et al.^13^ demonstrated similar results: rainbow trout fed Met restricted diets for 8 weeks showed increased fat accumulation in the liver and decreased fat accumulation in the muscle. Meanwhile, compared with the elevated Pi3k/Akt in the liver induced by the 0.40% diet, the protein levels of p-Pi3k and Akt in the muscle were both very low compared with those in the liver in the 0.40% and 0.84% Met dets. The results indicated that Met regulated lipogenesis was species-dependent in fish.

### Higher dietary Met levels (0.84% and 1.28%) induced fatty acid β-oxidation in both the liver and muscle of juvenile *Megalobrama amblycephala* than 0.40% diet

Unlike lipogenesis, β-oxidation is a process of fatty acid degradation, which supplies energy for the body. In the present study, higher dietary Met levels (0.84% and 1.28%) induced fatty acid β-oxidation in both the liver and muscle of *Megalobrama amblycephala*, which was demonstrated by the expression levels of *pparα* (except in muscle) and its downstream: *cpt1*^44^, were significantly upregulated by 0.84% and 1.28% Met compared with the 0.40% diet. Induced muscular *pparα* mRNA levels were found in the fish fed the 1.28% Met diet, higher than that in fish fed the 0.40% diet. Rolland et al.^7^ reported similar results in rainbow trout that hepatic *cpt1* expression levels in the low Met group were lower than those in the high Met group. In juvenile tiger puffer (*Takifugu rubripes*), lipolytic gene (*acox1* and *hsl*) expression levels were significantly induced by high dietary Met^9^. In the present study, 0.84% dietary Met increased hepatic *lp* mRNA levels compared with the 1.28% diet, and could catalyze triglyceride^45^. High Met preferentially improved muscular lipolysis, as evidenced by the muscular *lpl* mRNA level being induced by 1.28% Met in this study. The results of the present study implied that high dietary Met levels (0.84% or 1.28%) were more conducive to promoting lipolysis in the liver and muscle than the 0.40% dietary Met. These results also revealed the different lipolysis responses to dietary Met in the liver and muscle of juvenile blunt snout bream. The induced lipolysis and β-oxidation not only provide energy for the growth of juvenile blunt snout bream but also may partly contribute to the plasma TG and TC contents that did not show significant differences among the experimental groups. Similar results were also reported in juvenile silver pompano, *Trachinotus blochii* (Lacepede, 1801)^46^.

### Changes in glucose metabolism in the liver and muscle of *Megalobrama amblycephala* in response to dietary Met were dose-dependent

The liver, as the main tissue responsible for glucose homeostasis and plays a key role in regulating intermediary metabolism in response to nutritional status^47,48^. In the present study, the highest mRNA levels of *glut2* were found in the 1.28% Met diet, which promoted glucose transfer between blood and liver and glucose metabolism, which might be helpful for stable plasma glucose content^49^. Hepatic *gk*, *pfk* and *pk* relative mRNA expression levels were significantly induced by dietary 0.40% and/or 0.84% Met levels, suppressed by 1.28% dietary Met; while *pfk* and *pk* mRNA levels in the muscle were increased by the 1.28% diet compared with the control diet (0.84%). The present data about glycolysis revealed that lower dietary Met (0.40–0.84%) potentially promoted hepatic glucose utilization while muscular glucose utilization was enhanced by 1.28% dietary Met. Similar results were observed in cobia, in which 1.24% dietary Met enhanced hepatic glycolysis by increasing *pk* mRNA levels compared with an 0.70% diet^5^. Primary muscle cells of turbot treated with Met deprivation exhibited decreased *gk* and *pk* expression levels compared with the control^4^. The energy released by enhanced glycolysis in both the liver and muscle may contribute to the growth of blunt snout bream. In addition, the increased muscular glycolysis in the 1.28% diet may provide a substrate for lipid synthesis as shown in this study^50^. Additionally, in the current study, 0.40% dietary Met significantly induced hepatic *gk* and *pfk* expression levels compared with 1.28% Met level, while *pk* was not impacted. This result indicated that 0.40% Met potentially promoted the preparation stage of glycolysis but did not promote entry into the energy release stage^50^, which may be part of the reason that low dietary Met led to poor growth.

Regarding gluconeogenesis, another way of glucose metabolism, juvenile blunt snout bream fed 1.28% Met diet showed marked mRNA levels of the rate-limiting enzymes: *pepck* and *g6pase* in the liver. Also, the study was in line with the findings of Skiba-Cassy et al.^51^, who equally found that feeding rainbow trout with a high Met diet significantly enhanced the expression of hepatic *g6pase2* and *pepck* 2 h after a meal. And the results were also in agreement with Dai et al.^30^ and Lansard et al.^52^, also reported that high levels of amino acid could markedly up-regulate hepatic gluconeogenic gene mRNA levels in trout compared with those in fish treated with one-fold amino acid. Interestingly, combined with the hypothesis about glycolysis in the muscle described above, *Megalobrama amblycephala* fed 1.28% diet may activate the Cori cycle, that increased gluconeogenesis in the liver and resulting glucose is transported through the blood to the muscle where it is either utilized through glycolysis to supply energy demands of muscle contraction or build up muscle glycogen stores through glycogenesis^53,54^. The muscular glycogen synthesis was promoted by dietary Met supplementation (0.84% and 1.28%) that significantly increased muscular *gs* expression and glycogen contents in the present study. Higher dietary Met tended to enhance glucose and glycogen synthesis, which might be partly due to Met being a glucogenic amino acid^7^. In the muscle, 1.28% dietary Met markedly increased *pepck* mRNA relative expression levels in the current study to promote the production of phosphoenolpyruvate, which might help to activate PK and potentially link with lipid metabolism^50,53,55^.

## Conclusions

**Figure 6:**
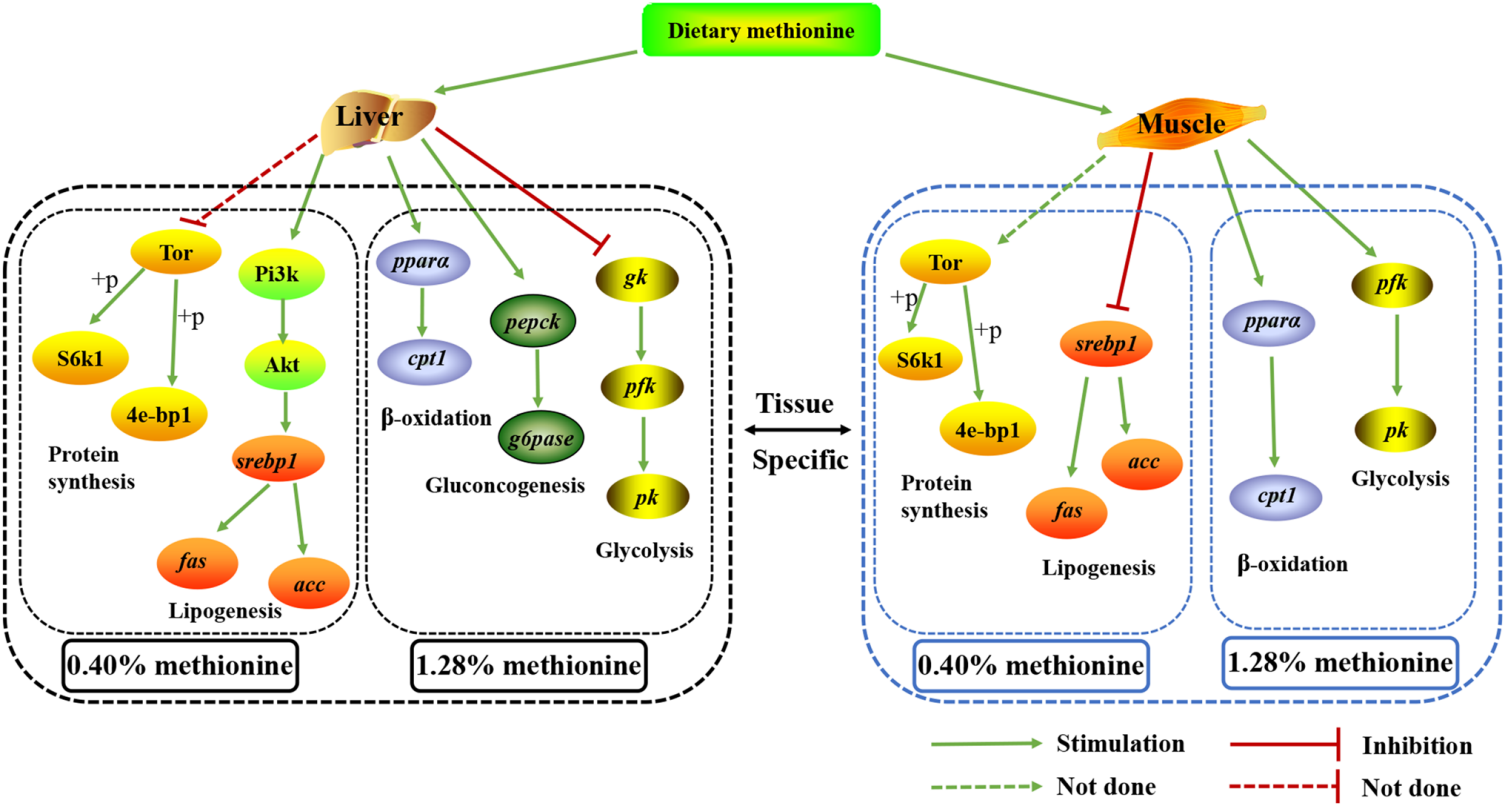
Scheme summarizing the nutrient metabolism in response to dietary methionine levels in juvenile *Megalobrama amblycephala*. Tor, target of rapamycin; 4e-bp1, eukaryotic initiation factor 4E binding protein-1; S6k1, ribosomal protein S6 kinase 1; Pi3k, phosphatidylinositol 3-kinase; Akt, protein kinase B; *srebp1*, *sterol regulatory element-binding protein 1*; *acc*, *acetyl-coa carboxylase*; *fas*, *fatty acid synthetase*; *cpt1*, *carnitine palmitoyl transferase 1*; *pparα*, *peroxisome proliferator activated receptor alpha*; *gk*, *glucokinase*; *pk*, *pyruvate kinase*; *pfk*, *phosphofructokinase*; *pepck*, *phosphoenolpyruvate carboxykinase*; *g6pase*, *glucose-6-phosphatase.*

In summary, this study revealed that 0.84% dietary Met could the enhance growth performance of juvenile blunt snout bream. Dietary Met levels had no significant effect on the body composition and plasma parameters of the fish. However, 0.40% Met level markedly increased hepatic lipid content in a Pi3k/Akt-*srebp1* independent TOR manner. 0.84–1.28% Met markedly increased the contents of lipid and glycogen in muscle by increasing related genes’ expression levels. The 0.40% dietary Met downregulated hepatic key TOR signaling genes, while improved muscular TOR signaling. The influence of nutrient metabolism in blunt snout bream in response to dietary Met levels in a tissue-specific and dose-specific manner (Fig. 6).

## Methods

### Ethical statement

All experimental protocols used in this study were approved by the Institutional Animal Care and Ethics Committee of Nanjing Agricultural University, Nanjing, China. [Permit number: SYXK (Su) 2011–0036]. All experiments on animals were performed following the Standardization Administration of China protocols and guidelines (GB/T 35892-2018). And the researchers declared that complied with the Animal Research: Reporting of In Vivo Experiments (ARRIVE) guidelines.

### Experimental design and diets

Given the dietary Met requirement of juvenile blunt snout bream was determined by Liao et al.^1^, three isonitrogenous (35% protein) and isoenergetic (18 kJ/g) feeds with the followed graded dietary Met levels were formulated: 0.40% (deficient), 0.84% (optimal, control) and 1.28% (excess). The composition of the basal feed was shown in Table 3. The composition and amino acid contents of the experimental diets (Table 4) are the same as those shown in our previous study^3^. It is worth noting that blunt snout bream, as an herbivorous fish, can digest plant protein well as fish meal^56,57^. Therefore, we used rapeseed meal and soybean meal as the main protein source in this study, which was to obtain more knowledge in practical situation, and to provide data support for practical production. As described in our previous study^1^, the pellet diets were processed by F-26 (II) (South China University of Technology, China), air-dried, and finally stored in a refrigerator at − 20 °C until feeding.

**Table 3:** Ingredient and proximate composition of basal diets (% dry basis).

Ingredients (%)			
Fish meal	2	Choline chloride	0.1
Rapeseed meal	12	Ethoxy quinoline	0.01
Soybean meal	30	Bentonite	2
Wheat meal	28	Amino acid premix	5.72
Rice bran	12.78	Glycine	0.84
Soybean oil	1.5	l-Methionine	0^a^
Soybean phospholipid	1	**Proximate composition**	
Vitamin C	0.05	Protein (%)	35
Vitamin and mineral premix	1	Lipid (%)	8
Monocalcium phosphate	3	Energy (KJ/g)	18.7

The basal diet in this experiment was referred to our previous study^3^.

^a^l-Methionine supplementation (0, 0.42%, 0.84%) was used to meet the graded methionine levels (0.40%, 0.84%, 1.28%), and Glycine was used to balance methionine supplementation.

**Table 4:** Amino acids composition of ingredient (% dry basis).

Amino acids	2%	12%	30%	28%	12.78%	CAPP^f^	Total	34% WBP^g^
	FM^a^	RM^b^	SM^c^	WM^d^	RB^e^			
**EAA**^**h**^								
Arginine	0.08	0.30	1.12	0.14	0.15	0.22	2.01	2.01
Histidine	0.03	0.13	0.40	0.08	0.05	0.07	0.76	0.76
Isoleucine	0.06	0.20	0.72	0.13	0.07	0.33	1.49	1.49
Leucine	0.10	0.35	1.18	0.25	0.13	0.40	2.40	2.40
Lysine	0.10	0.27	0.94	0.08	0.09	0.96	2.44	2.44
Methionine	0.04	0.10	0.20	0.06	0.04	Variable	0.43	0.90
Phenylalanine	0.05	0.20	0.80	0.17	0.09	0.16	1.47	1.47
Threonine	0.05	0.21	0.60	0.10	0.07	0.38	1.41	1.41
Valine	0.06	0.25	0.74	0.15	0.10	0.26	1.57	1.57
Tryptophan	0.01	0.07	0.21	0.04	0.02	0.00	0.35	0.32
**NEAA**^**i**^								
Aspartic acid	0.12	0.35	1.77	0.15	0.17	0.28	2.84	2.84
Serine	0.05	0.21	0.78	0.17	0.08	0.16	1.45	1.45
Glycine	0.10	0.25	0.66	0.13	0.10	1.24	Variable	2.50
Alanine	0.09	0.22	0.66	0.11	0.11	0.96	2.15	2.15
Cystine	0.01	0.12	0.22	0.08	0.04	0.00	0.47	0.22
Gulmatic acid	0.18	0.84	2.78	1.14	0.26	0.00	5.19	4.64
Proline	0.06	0.31	0.78	0.40	0.08	0.28	1.92	1.92

^a^*FM* fish meal.

^b^*RM* rapeseed meal.

^c^*SM* soybean meal.

^d^*WM* wheat meal.

^e^*RB* rice bran.

^f^*CAAP* crystalline amino acid premix.

^g^*WBP* whole-body protein.

^h^*EAA* essential amino acid.

^i^*NEAA* non-essential amino acid.

### Experimental fish and feeding

As mentioned in our previous study^3^, the experimental fish were obtained from the breeding farm of Freshwater Fisheries Research Center (FFRC) of Chinese Academy of Fishery Sciences (Wuxi, Jiangsu, China). After a 15-day acclimation period, the juvenile fish consistent with health and specification (initial weight 4.37 ± 0.01 g) were randomly distributed into nine nylon cages in the pond, and every cage (1 m × 1 m × 1 m) with 20 fish. Each diet was randomly assigned to triplicate cages. Fish were hand-fed three times daily at 7:30, 12:00 and 16:30 for 75 days, until apparent satiation based on visual observation of fish feeding behavior. The water quality was tested weekly (ProDSS Multiparameter Water Quality Meter, YSI, USA), the water temperature was maintained at 28 to 31 °C, pH was maintained from 7.0 to 7.8, ammonia nitrogen was not higher than 0.05 mg/L and dissolved oxygen was higher than 6.0 mg/L. And the photoperiod was the same as natural light (12 h:12 h).

### Sampling

24 h after the fish were fasted, the total weight and number of fish in every cage were counted to calculate the relative growth indices at the end of the experiment. Five fish from every cage were anesthetized by using MS-222 (100 mg/L), and then to collect blood from the caudal vein with disposable medical syringes. After drawing blood, the abdominal cavity of the fish was cut from the cloaca along the lateral line, and then the liver was sampled. And muscle was taken from the dorsal white muscle. Plasma was obtained by centrifugation of blood samples (3500 × g, 10 min, 4 °C). The other two fish from each cage were collected to test whole body composition. The samples were stored at -80 °C until analyzed.

### Analyses of composition and amino acids

The content of moisture, crude protein and lipid, and ash of feeds, whole body and ingredients were analyzed according to the methods described in AOAC^58^. The lipid contents in the liver and muscle were extracted by using chloroform: methanol (C–M) (2:1, v/v) according to the methods described in Peng et al.^59^. The concentrations of amino acid in feeds and ingredients were analyzed by using an amino acid analyzer (SYKAM S-433D, Sykam GmbH, Munich, Germany).

### Analyses of plasma parameters and glycogen contents

Plasma GLU, TC, and TG were tested by using Mindray BS-400 automatic biochemical analyzer (Mindray Medical International Ltd., Shenzhen, China), and the assay kits (GLU: GL9720; TC: CH8727; TG: TR7734) purchased from Shanghai Zhicheng Biological Technology Co. Ltd (Shanghai, China). Glycogen contents in the liver and muscle were tested by kits (A043-1-1) purchased from Nanjing Jiancheng Bioengineering Institute (Nanjing, China).

### Quantitative real-time PCR (qRT-PCR)

Total RNA from the liver and muscle from three fish in each cage was extracted by RNAiso Plus (Cat# 9109, Takara, Baobio Engineering (Dalian) Co., Ltd, Dalian, China). The quality and quantity of RNA were tested by a spectrophotometer (Thermo Fisher Multiskan GO, Shanghai, China). Finally, qRT-PCR was performed according to the instructions of One Step TB Green PrimeScript Plus RT-PCR Kit (Cat# RR096A, Takara, Baobio Engineering (Dalian) Co., Ltd, Dalian, China) on a CFX96 Touch Real-Time PCR Detection System (BIO-RAD, California, USA). Specific primers of target genes (Table 5) were designed according to the partial cDNA sequences showed in Gao et al.^60^. The assay used β-actin as the reference gene, and the target gene expression levels were analyzed using the 2^−∆∆ct^ model.

**Table 5:** Primer sequence for qRT-PCR.

Target gene	Primer sequence	
	Forward (5′–3′)	Reverse (3′–5′)
*tor*^a^	TTTACACGAGCAAGTCTACGGA	CTTCATCTTGGCTCAGCTCTCT
*4e-bp1*^b^	GCTGGCTGAGTTTGTGGTTG	CGAGTCGTGCTAAAAAGGGTC
*s6k1*^c^	GGTGCATGTCACCTTATGGG	AGCTGGCAGCACTTCTAGTC
*gk*^d^	GCTTCCACTGGGATTCACCT	CGACGTTATTGCCTTCAGCG
*pk*^e^	CGAGATTGAGAACGGAGGCA	GTCCTTCTCAGACACTGCGG
*pfk*^f^	TAGGATCAAGCAATCCGCCG	CCTGCCATGGTTGCCAGATA
*pepck*^g^	TCGCCTGGATGAAGTTCGAC	GTCTTGGTGGAGGTTCCTGG
*g6pase*^h^	TTCAGTGTCACGCTGTTCCT	TCTGGACTGACGCACCATTT
*fbp*^i^	CGGCAGCCCATTATCATTGC	GCGTACACTGGACTCTCCAC
*gs*^j^	TTACACGGTCATTGCGTCCA	GACACAGCTCAGTCGGTGAA
*g6pd*^k^	TGGAGAAACCTTTTGGCCGT	CTGGGTACCAAACGGCTCTT
*glut2*^l^	CGGTGAAACCGAACAGGAGT	TTCTTTGAGATCGGGCCTGG
*glut4*^m^	CCATTGCTGAGCTCTTTCGC	GCGTACACTGGACTCTCCAC
*acc*^n^	TAGCAGTGAGCATTGGCACA	CATCGCTGGCGTATGAGGAT
*fas*^o^	GTTTGCCAACCGCTTGTCTT	GGCCATGGCGAATAGCATTG
*srebp1*^p^	ACAACAGTAGCGACACCCTG	AGGAGCGGTAGCGTTTTTCA
*cpt1*^q^	CAAGCTCTGAGGGCCAAAGG	TGTACCATCGAGGCCGTTTC
*pparα*^r^	CGTTGACGTCCTTCTCTGCT	ATGTCCCACAACGCTATCCG
*lpl*^s^	GCCACGAGTGTTGGTGTGAA	TGGCCCTAGCTTTGAGTACG
*lp*^*t*^	GTTTCTGGATTTGGGTCG	TCTGATGGGATCTGGCAC
β-actin	TCGTCCACCGCAAATGCTTCTA	CCGTCACCTTCACCGTTCCAGT

^a^*tor*, *target of rapamycin.*

^b^*4e-bp1*, *eukaryotic initiation factor 4e binding protein-1.*

^c^*s6k1*, *ribosomal protein s6 kinase 1.*

^d^*gk*, *glucokinas.*

^e^*pk*, *pyruvate kinase.*

^f^*pfk*, *phosphofructokinase.*

^g^*pepck*, *phosphoenolpyruvate carboxykinase.*

^h^*g6pase*, *glucose-6-phosphatase.*

^i^*fbp*, *fructose-1,6-biphosphatase.*

^j^*gs*, *glycogen synthase.*

^k^*g6pd*, *glucose-6-phosphate dehydrogenase.*

^l^*glut2*, *glucose transporters 2.*

^m^*glut4*, *glucose transporters 4.*

^n^*acc*, *acetyl-coa carboxylase.*

^o^*fas*, *fatty acid synthetase.*

^p^*srebp1*, *sterol regulatory element-binding protein 1.*

^q^*cpt1*, *carnitine palmitoyl transferase 1.*

^r^*pparα*, *peroxisome proliferator activated receptor alpha.*

^s^*lpl*, *lipoprotein lipase.*

^t^*lp*, *lipase.*

### Western blot analysis

50 mg liver and muscle samples from one fish in each cage were rinsed twice with ice-cold PBS, the PBS was removed by centrifugation, and then the tissues were put into ice-cold RIPA buffer (50 mM Tris, pH 8.0; 150 mM NaCl; 1% TritonX-100; 0.1% SDS; 1% sodium deoxycholate; 1 mM EDTA) with 5 mM NaF, 2 mM Na_4_P_2_O_7_, 2 mM β-glycerophosphate, 1 mM Na_3_VO_4_, 0.1 mmol/L PMSF, and protease and phosphatase inhibitor cocktail (RFT194, Biolab). The supernatant was collected after centrifugation at 12,000 × g for 10 min at 4 °C. The protein contents were tested by the BCA method (BB-3401, BestBio). Quantified samples were added to prefabricated SDS-PAGE gels for electrophoresis and then transferred to NC membrane (66,485, Bio Trace). The blot was blocked with 5% nonfat dry milk in TBST for 2 h at room temperature. After overnight incubation of the membrane and primary antibodies, the membrane was incubated for 1 h with appropriate secondary antibodies. A Beyo ECL Star kit (Beyotime Biotechnology) was used to develop the signal. The bends were scanned and quantified using a chemiluminescence imaging system (Clinx, Shanghai, China). Antibodies against the following proteins were used: phospho-phosphatidylinositol 3-kinase (p-Pi3k, Tyr458/Tyr199) (Cat# 4228), phospho-eukaryotic initiation factor 4E binding protein-1 (p-4e-bp1, Thr37/46) (Cat# 9459), and 4e-bp1 (Cat# 9452) were purchased from Cell Signaling Technology Inc. Pi3k (Cat# 20,584–1-AP), ribosomal protein S6 kinase 1 (S6k1, Cat# 14,485–1-AP) and protein kinase B (Akt, Cat# 10,176–2-AP) were purchased from Proteintech Group, Inc. And β-actin (Cat# AY0573) was purchased from Abways Technology. Among them, antibodies Pi3k, S6k1 and Akt were successfully used in our previous study^16^. The densities of the protein bands were normalized to that of β-actin, which served as an internal control. In addition, the need is, the blots were cut prior to hybridization with antibodies, therefore, original images of full-length blots cannot be provided.

### Statistical analysis

Parameters were calculated as followed:

$$Feed\;conversion\, ratio\;(FCR) = \frac{dry\;feed\;fed\;(g)}{{wet\;weight\;gain\;(g)}}$$

$$Specific\;growth\;rate\;(SGR) = 100 \times \frac{{\left[ {In\left( {final\;fish\;weight} \right) - In\left( {initial\;fish\;weight} \right)} \right]}}{the\;experimental\;duration\;in\;days}$$

$$Weight\;gain\;rate\;(WGR) = 100 \times \frac{final\;weight\;(g) - initial\; weight\;(g)}{{initial\; weight \;(g)}}$$

Data were analyzed by one-way analysis of variance (ANOVA) and Tukey's multiple comparisons with SPSS 16.0 software. The results are presented as the means with SEM and *P* < 0.05 indicates a statistical significance.

### Equipment and settings

Figures 1, 2, 3b,c,d, 4 and 5 were produced using Prism 5 software. Figure 6 was drawn with PowerPoint software. Finally, the resolution and size of all figures were performed using photoshop CC software. Western blot images were acquired using the chemiluminescence imaging system (Clinx ChemiScope 6200, Shanghai) and auto-exposure settings. Clinx chemical analysis software was used to auto-analyze the gray value of the western blot.

## Supplementary Information

Supplementary Information.

## Data Availability

All data generated or analyzed during this study are included in this published article.
