# Supplementary material for: Nutrient metabolism in the liver and muscle of juvenile blunt snout bream (Megalobrama amblycephala) in response to dietary methionine levels
Source: Sci Rep. 2021 Dec 13;11:23843. doi: 10.1038/s41598-021-03084-3 (PMC8668952; doi:10.1038/s41598-021-03084-3)
Supplement: Supplementary file 1 — Supplementary Information. [file 41598_2021_3084_MOESM1_ESM.docx]

**Supplementary Information**

Nutrient metabolism in the liver and muscle of juvenile blunt snout bream (*Megalobrama amblycephala*) in response to dietary methionine levels

**Ke Ji^1^, Hualiang Liang^2^, Mingchun Ren^1,2*^, Xianping Ge^1,2*^, Liangkun Pan^2^, Heng Yu^1^**

^1^Wuxi Fisheries College, Nanjing Agricultural University, Wuxi 214081, China.

^2^Key Laboratory for Genetic Breeding of Aquatic Animals and Aquaculture Biology, Freshwater Fisheries Research Center (FFRC), Chinese Academy of Fishery Sciences (CAFS), Wuxi 214081, China.

Due to the blots were cut prior to hybridization with antibodies, therefore, original images of full-length blots cannot be provided. The original blots and multiple exposure images were supplied as the Supplementary Material below.


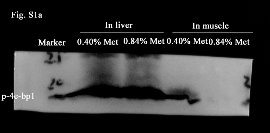

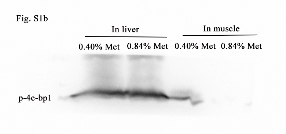

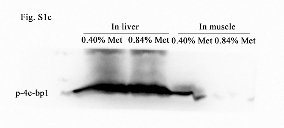

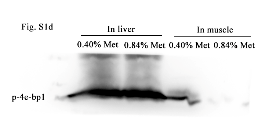


**Fig. S1** the original blots and multiple exposures of p-4e-bp1 in the liver and muscle of fish fed 0.40% Met diet and 0.84% Met diet.


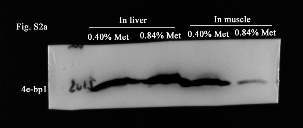

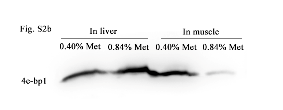

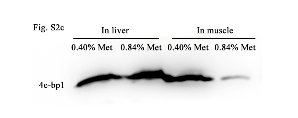

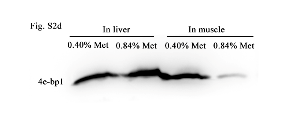


**Fig. S2** the original blots and multiple exposures of 4e-bp1 in the liver and muscle of fish fed 0.40% Met diet and 0.84% Met diet.


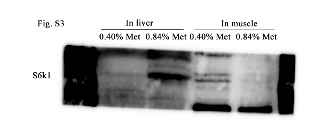


**Fig. S3** the original blots and exposures of S6k1 in the liver and muscle of fish fed 0.40% Met diet and 0.84% Met diet.


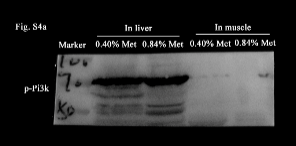

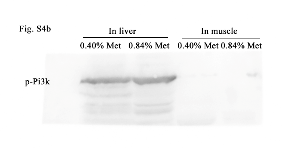

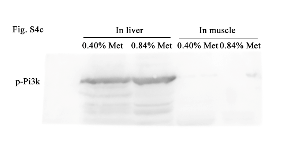

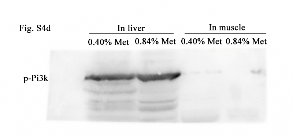

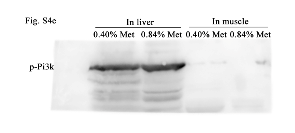

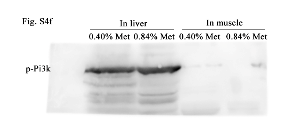

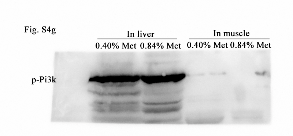


**Fig. S4** the original blots and multiple exposures of p-Pi3k in the liver and muscle of fish fed 0.40% Met diet and 0.84% Met diet.


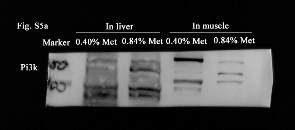

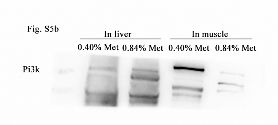

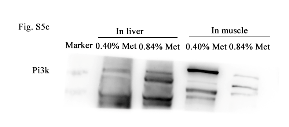

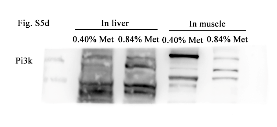

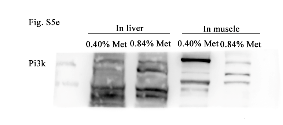

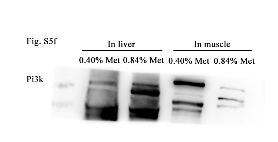


**Fig. S5** the original blots and multiple exposures of Pi3k in the liver and muscle of fish fed 0.40% Met diet and 0.84% Met diet.


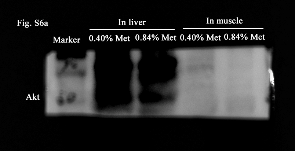

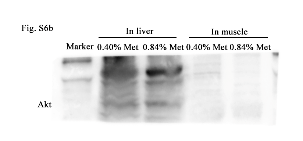

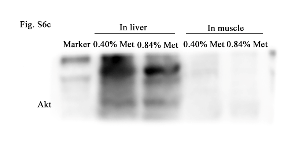

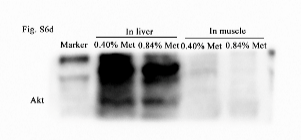

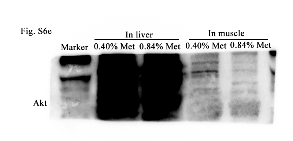


**Fig. S6** the original blots and multiple exposures of Akt in the liver and muscle of fish fed 0.40% Met diet and 0.84% Met diet.


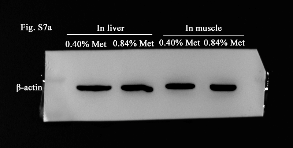

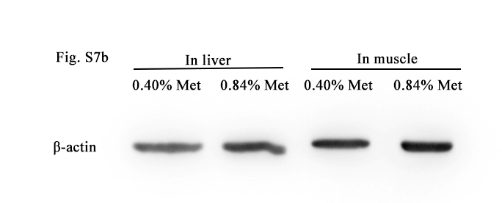

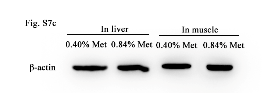

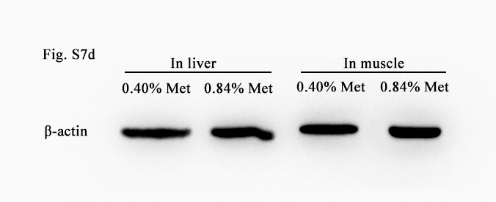


**Fig. S7** the original blots and multiple exposures of β-actin in the liver and muscle of fish fed 0.40% Met diet and 0.84% Met diet.
